# Effects of *Trichinella spiralis* and its excretory/secretory products on autophagy of host muscle cells *in vivo* and *in vitro*

**DOI:** 10.1371/journal.pntd.0009040

**Published:** 2021-02-18

**Authors:** Xiaoxiang Hu, Xiaolei Liu, Xue Bai, Li Yang, Jing Ding, Xuemin Jin, Chen Li, Yulu Zhang, Yanfeng Li, Yong Yang, Mingyuan Liu

**Affiliations:** 1 Key Laboratory of Zoonosis Research, Ministry of Education, Institute of Zoonosis, College of Veterinary Medicine, Jilin University, Changchun, China; 2 Jiangsu Co-innovation Center for Prevention and Control of Important Animal Infectious Diseases and Zoonoses, Yangzhou, China; NIH-National Institute for Research in Tuberculosis-ICER, INDIA

## Abstract

*Trichinella spiralis* (*T*. *spiralis*) is a widely distributed pathogenic microorganism that causes trichinellosis, a disease that has the potential of causing severe harm to their host. Numerous studies have demonstrated that autophagy can be triggered by microbial infection, such as bacteria, viruses, protozoa, and parasitic helminths. However, it’s still unknown whether autophagy can facilitate host resistance to *T*. *spiralis* infection. The present study examined the role of autophagy in striated muscle cell transformation following infection with *T*. *spiralis* in BALB/c mice. Transmission electron microscopy (TEM) was used to detect the production of the host diaphragm autophagosome after *T*. *spiralis* infection, and changes in the protein and transcriptional levels of autophagic marker proteins were also detected. The significance of autophagy in *T*. *spiralis* infection, namely inhibition of *T*. *spiralis* growth, was preliminarily evaluated by conducting *in vivo* experiments using autophagy inhibitors. Besides, we studied the effect of excretory-secretory products (ES) of *T*. *spiralis* on autophagy of C2C12 myoblasts. The changes in protein and gene expression levels in autophagy-related pathways *in vitro* and *in vivo* were measured as further evidence. The results showed that *T*. *spiralis* infection induced autophagy in the host muscle cells. Meanwhile, ES inhibited autophagy of myoblasts *in vitro*, but this did not affect the cell viability. The upregulation and downregulation of autophagy-related factors in skeletal muscle cells may indicate an adaptive mechanism providing a comfortable niche for the parasite.

## Author summary

Autophagy, a intracellular degradation system, is a kind of unique phenomenon in eukaryotic cells. The commonly referred autophagy is the process of forming autophagosomes by wrapping the cytoplasmic components with double-membrane structure, and then fusing with lysosomes to degrade the internal substances of the cell. Autophagy can be induced by various pathogens including parasites. When the body is infected with intracellular parasites, the host cell can remove the parasites by autophagy. However, parasites have also evolved defence mechanisms that use autophagy in host cells to promote growth. These can be seen in some intracellular parasitic infections such as *Toxoplasma gondii* and *Plasmodium*. Although the role of autophagy in other parasitic infections has been revealed, it remains unclear whether autophagy is involved in the invasion process by *Trichinella*. We investigated the role of *Trichinella* infection on host muscle cells autophagy and the effect of autophagosome formation on the survival of *T*. *spiralis*. Understanding the role of autophagy in the interaction between parasitic infection and host cell is of great significance for the prevention and treatment of *Trichinella* infection and the development of anti-parasite drugs.

## Introduction

Trichinellosis is a widespread and severe foodborne parasitic disease caused by the consumption of raw, undercooked pork, or its products containing infective larvae of *Trichinella* nematodes [1]. The infection spectrum of *Trichinella* is extensive; it can invade more than 150 animals, including humans [2]. Trichinellosis not only affects human health but also increases food safety risks, thus causing substantial economic losses. As such, there is a need to understand the pathogenesis of trichinellosis, and thereby prevent disease occurrence and progression.

After *Trichinella* infection, the infective muscle larvae (ML) molt into adulthood, mate, and reproduce. Subsequently, the gravid females release new-born larvae (NBL), which migrate and invade skeletal myocytes. The NBL grow rapidly, cause extensive damage to the host myocytes, and transform them into nurse cell that nourishes and protects the parasites from the host immune response [3,4]. ES of *T*. *spiralis* are a complex mixture of different molecules with various biological activities. These molecules are essential for the long-term survival of *T*. *spiralis*, as they facilitate the evasion and modulation of host immunity [5]. The effects of *T*. *spiralis* on host cells and molecules occur through ES. Muscle modifications induced by injection of ML-ES are similar to those produced during ML development, suggesting that *T*. *spiralis* uses ES to cause its effects in muscle to allow nurse cell development [6].

Autophagy is a fundamental metabolic process that aid in the degradation of non-essential or dysfunctional cell components or exogenous microorganisms via the lysosomal system to achieve cell homeostasis and protection [7]. Of note, this phenomenon can occur in normal physiological and pathological processes. Three forms of autophagy have been described so far, namely, macroautophagy, chaperone-mediated autophagy, and microautophagy [8]. Macroautophagy, as the most common autophagic process with an immense research interest, is discussed in the present study extensively. Macroautophagy is regulated by several autophagy-related genes (Atg) and associated enzymes. The key protein in autophagy is microtubule-associated protein 1 light chain 3 (LC3), a ubiquitin-like regulator of autophagosome biogenesis [9]. The mammalian target of rapamycin (mTOR) kinase is a critical molecule in autophagy induction. Akt and MAPK signaling pathways inhibit autophagy by activating the mTOR pathway. Conversely, the AMPK signaling pathway promotes autophagy via negative regulation of the mTOR pathways. Meanwhile, phosphorylated ULK1 is an essential regulator of autophagy. Recent evidence indicates that AMPK and mTOR kinases catalyze the phosphorylation of ULK1 under starvation and adequate nutrition, respectively, which play a very crucial role in autophagy. During autophagy, a portion of the cytoplasm is enclosed by an isolating membrane, or "phagophore", thus forming a double-membrane structure called an autophagosome. The outer membrane of the autophagosome fuses with the lysosome to form an autolysosome, after which lysosomal enzymes degrade the autophagosomal contents. As more autophagosomes and lysosomes fuse, autolysosomes enlarge; and lysosomes are fragmented to provide cellular energy and usable substances for cells [10]. Although the primary role of autophagy is the provision of new energy in response to nutrient stresses, the process also plays a housekeeping role by removing misfolded or aggregated proteins and clearing damaged organelles [11]. Recent studies have implicated a crucial role for the autophagy pathways and related proteins in immunity and inflammation. Specifically, they have been shown to balance the beneficial and detrimental effects of immunity and inflammation and thereby may protect against infectious, autoimmune, and inflammatory diseases [8].

In addition to destroying exogenous microscopic organisms, such as viruses and bacteria, autophagy occurs during parasitic infections and exhibits specific unique characteristics. Initial observations in activated macrophages described autophagosome-like double-membrane vacuoles surrounding entire *Toxoplasma gondii*, suggesting that autophagy could be involved in the elimination of the tachyzoites [12]. On the contrary, reduced parasite growth was observed in Atg5-deficient cells, implying that autophagy could also promote parasite development. Host cell autophagy induced by *Toxoplasma gondii* and *Plasmodium* is exploited by the parasites to acquire nutrients, which contributes to their growth and development [13,14]. Recent studies have also revealed that the inhibition of autophagy or excess autophagy can induce cell apoptosis in *Echinococcus granulosus* [15].

Although the role of autophagy in other parasitic infections has been somewhat unmasked, it is still unclear whether autophagy is involved in the invasion of skeletal muscle cells by *Trichinella*. Also, the specific roles of autophagy in the invasion process are not known. This study aimed to investigate the role of autophagy in *Trichinella* infection and the effect of autophagosome formation on the survival of intracellular *Trichinella* in skeletal muscle cells. Understanding the role of autophagy in the interaction between parasitic infection and host cell is of great significance for the prevention and treatment of *Trichinella* infection and the development of novel anti-parasite drugs.

## Materials and methods

### Ethics statement

All experiments were conducted according to the regulations of the Administration of Affairs Concerning Experimental Animals in China. The procedure of animal experiments was approved by the Institutional Animal Care and Use Committee of Jilin University (Permit No. 20170318).

### Animals and *T*. *spiralis* infection

Sprague Dawley rats and BALB/c mice were obtained from the Experimental Animal Center of College of Basic Medical Sciences, Jilin University (Changchun, China). To examine the occurrence of autophagy, the mice were orally infected with 500 ML of *T*. *spiralis* (ISS534) each and were sacrificed at 0, 7, 14, 21 and 28 days post infection (dpi) respectively. Additionally, 27 mice were divided into three groups: the control, low dose (5 μg/g), and high dose (10 μg/g) groups. Each mouse was intraperitoneally injected with autophagy inhibitor 3-Methyladenine (3-MA, Sigma-Aldrich) dissolved in 100 *μ*L of preheated double distilled water or water alone, every other day until 28 dpi.

### Histopathological staining

Diaphragms collected from *T*. *spiralis*-infected mice were fixed in 10% paraformaldehyde, embedded in paraffin and then sliced. Finally, these sections were stained with hematoxylin and eosin and blindly observed under a microscope (IX73, Olympus, Japan).

### ML-ES preparation

Muscle larvae ES were collected from experimentally infected SD rats at 30 dpi, as per the methods described previously [16]. Briefly, following recovery and washing repeatedly in sterile saline, the ML were incubated in prewarmed serum-free RPMI 1640 medium containing 100 U/ml penicillin and 100 μg/ml streptomycin at 37°C with 5% CO_2_ for 24 h. Next, the supernatant was collected and concentrated 100-fold on a YM-3 membrane (Amicon, Beverly, MA) at 4°C, then dialyzed against saline solution [17]. The protein concentration was measured using the BCA protein assay kit (GenStar, China). Finally, ML-ES was aliquoted and kept at −80°C till use.

### C2C12 cell culture

The C2C12 cell line (ATCC, Manassas, VA, USA) was seeded into 24- or 6-well plates at 1×10^5^ or 1×10^6^ cells per well, respectively. The cells were maintained in DMEM medium supplemented with 10% fetal bovine serum, penicillin, and streptomycin at 37°C with 5% CO_2_. In the differentiation medium, 2% horse serum (GIBCO, USA) was used in place of fetal bovine serum. After that, the cells were co-cultured with or without ML-ES, Rapamycin (Rapa, Cat. No.: HY-10219, MedChemExpress, USA), or 3-MA for different durations at specific concentrations.

### Transmission electron microscopy

Small pieces of the diaphragm were isolated and fixed in 0.1 M sodium cacodylate-buffered 2.5% glutaraldehyde solution at 4°C and rinsed with 0.1 M sodium cacodylate-buffered 7.5% sucrose. Next, 1% osmium tetroxide (OsO4) in 0.033 M veronal acetate buffer containing 4% sucrose was added for post-fixation treatment at 4°C. The tissue samples were washed (3×10 min) with 0.05 M veronal acetate buffer containing 6% sucrose and treated at room temperature with ethanol 100%/EMbed 812 mixture without DMP-30 (1:1) for 2 h, then with ethanol 100%/EMbed 812 combinations without DMP-30 (2:3) overnight. Afterward, tissue samples were treated at room temperature with EMbed 812 resin mixture (without DMP-30) for 2×2 h, followed by EMbed 812 resin mixture (containing DMP-30) for 1 h. Tissues were then transferred into a gelatin capsule, covered with EMbed 812 resin mixture containing DMP-30, and polymerized at 60°C for approximately 36 h. Ultrathin sections were obtained with an ultramicrotome using a standard diamond knife (Element six), captured and air-dried on 200 mesh copper grids. Sections were stained with 2% uranyl acetate (in water) for 15 min in the dark and rinsed with ultrapure water. Once again, sections were stained with Reynolds solution (pH 12.4) for 10 min and rinsed with 0.05 M NaOH Titrisol and CO_2_-free ultrapure water. Samples were viewed using a TEM at an accelerating voltage of 80 kV.

### Western blot

Total proteins from cells or diaphragms were extracted using the Minute Total Protein Extraction Kit (Invent Biotechnologies, Inc, China) as per the user manual. The protein concentrations were determined by the BCA protein assay kit, and equal amounts of the protein (40 μg) were fractionated on 12% sodium dodecyl sulphate–polyacrylamide gel electrophoresis (SDS-PAGE). The resolved bands were then transferred onto a polyvinylidene difluoride (PVDF) membrane (Roche, Germany). The membrane was blocked with 5% (*w/v*) non-fat dry milk in PBST (0.05% Tween-20 in PBS) for 2 h at room temperature (RT), and then incubated with the following primary antibodies overnight: Anti-GAPDH antibody (ab8245, Abcam, UK), Anti-LC3B antibody (ab192890, Abcam, UK), Anti-SQSTM1/p62 antibody (ab109012, Abcam, UK), Anti-Beclin-1 antibody (ab207612, Abcam, UK), p38 MAPK Antibody (AF6456, Affinity Biosciences, China), Phospho-p38 MAPK (Thr180/Tyr182) Antibody (AF4001, Affinity Biosciences, China), Erk1/2 Antibody (AF0155, Affinity Biosciences, China), Phospho-Erk1/2 (Thr202/Tyr204) Antibody (AF1015, Affinity Biosciences, China), JNK1/2/3 Antibody (AF6318, Affinity Biosciences, China), Phospho-JNK1/2/3 (Thr183+Tyr185) Antibody (AF3318, Affinity Biosciences, China), AMPKα (23A3) Rabbit mAb (#2603, Cell Signalling Technology, USA), Phospho-AMPKα (Thr172) (40H9) Rabbit mAb (#2535, Cell Signalling Technology, USA), Akt (pan) (C67E7) Rabbit mAb (#4691, Cell Signalling Technology, USA), Phospho-Akt (Ser473) (D9E) XP Rabbit mAb (#4060, Cell Signalling Technology, USA), PI3 Kinase Class III (D4E2) Rabbit mAb (#3358, Cell Signalling Technology, USA), Phospho-4E-BP1 (Thr37/46) (236B4) Rabbit mAb (#2855, Cell Signalling Technology, USA), Anti-ULK1 antibody (ab128859, Abcam, UK), Phospho-ULK1 (Ser757) (D7O6U) Rabbit mAb (#14202, Cell Signalling Technology, USA), mTOR (7C10) Rabbit mAb (#2983, Cell Signalling Technology, USA), Phospho-mTOR (Ser2448) (D9C2) XP Rabbit mAb (#5536, Cell Signalling Technology, USA). After that, the membrane was washed five times with PBST and incubated with an Anti-rabbit IgG, HRP-linked Antibody (#7074, Cell Signalling Technology, USA) in blocking buffer for 2 h at RT. Signals were detected using the Pierce ECL Western Blotting Substrate (ThermoFisher Scientific, USA) and UVP Chemstudio (Analytik Jena, Germany) according to the manufacturers’ instructions.

### Quantitative real-time PCR

Total RNA were extracted from the C2C12 myoblasts and diaphragm samples using the RNAiso Plus (TaKaRa) kit, according to the manufacturer’s instructions. The quality and concentration of the total RNA were measured using a NanoDrop 2000 spectrophotometer (ThermoFisher Scientific, USA). The RNA were then reverse transcribed to cDNA using the QuantiNova Reverse Transcription Kit (QIAGEN, Germany), as per the supplier’s recommendations. The resulting cDNA were analysed by qRT-PCR using an Applied Biosystems StepOnePlus Real-Time PCR System (ThermoFisher Scientific, USA). The primer sequences used in this study are listed in Table 1. Each sample was assayed in triplicate wells on the same plate, and each reaction contained FastStart Essential DNA Green Master (Roche, Germany), forward primer (0.2 μM), reverse primer (0.2 μM), and cDNA template, and topped up to 20 μl with nuclease-free water. Amplification of all genes was performed under the following conditions: a holding time of 95°C for 10 min, followed by 40 cycles of denaturation at 95°C for 15 s, primer annealing at 59°C for 30 s. Data of mRNA fold expression values were analyzed using the relative CT (2^-ΔΔCt^) method [18,19].

**Table 1:** The primer sequences used for qRT-PCR.

Gene	Primer sequence (5′→3′)
GAPDH	forward 5′-TACCCCCAATGTGTCCGTC-3′
	reverse 5′-AAGAGTGGGAGTTGCTGTTGAAG-3′
LC3B	forward 5′-CCACCAAGATCCCAGTGATTAT-3′
	reverse 5′-TGATTATCTTGATGAGCTCGCT-3′
p62	forward 5′-GAACACAGCAAGCTCATCTTTC-3′
	reverse 5′-AAAGTGTCCATGTTTCAGCTTC-3′
Beclin-1	forward 5′-TAATAGCTTCACTCTGATCGGG-3′
	reverse 5′-CAAACAGCGTTTGTAGTTCTGA-3′
ULK1	forward 5′-ACTCAGGTGCACAATTACCAG-3′
	reverse 5′-CTTGGGGAGAAGGTGTGTAG-3′
Bcl-2	forward 5′-GATGACTTCTCTCGTCGCTAC-3′
	reverse 5′-GAACTCAAAGAAGGCCACAATC-3′
Atg5	forward 5′-AGTCAAGTGATCAACGAAATGC-3
	reverse 5′-TATTCCATGAGTTTCCGGTTGA-3′
Atg9a	forward 5′-GCCTGGTGCTGTCCGAATACG-3′
	reverse 5′-GCCTGGGTCTGCTGCTTGTG-3′
Atg12	forward 5′-GCCTCGGAACAGTTGTTTATTT-3′
	reverse 5′-CAGTTTACCATCACTGCCAAAA-3′
Atg14	forward 5′-GCGTGTAAGCGATGAGGAGACTG-3′
	reverse 5′-CCTGGGTGCTCTGGCTCTGG-3′
DRAM1	forward 5′-GCATCGTAGCCAACTTCCAGGAG-3′
	reverse 5′-ATGATCGATTGCAGGAGCGTGTAC-3′
ZFYVE1	forward 5′-ACATCTGCCTCCACTGACTCTCC-3′
	reverse 5′-TCCGCACCACCGTATCCACAG-3′
VMP1	forward 5′-TAAGGATCAGCACAATGGAAGT-3′
	reverse 5′-TCCAGAGAGAAATACTGCAAGG-3′

### Immunocytofluorescence

After treatment in 24-well plates with ES, Rapa or 3-MA, the coverslips were washed with 0.01 M PBS, and the cells were then fixed in pre-chilled 4% (*w/v*) paraformaldehyde for 15 min. The coverslips were permeabilized with 0.1% Triton X-100 in PBS for 10 min and then blocked with 5% (*v/v*) goat serum in PBS for 2 h. Next, cells were incubated overnight at 4°C with the primary antibody Anti-LC3B antibody, Anti-SQSTM1/p62 antibody, and Anti-Beclin-1 antibody. The coverslips were washed thrice in 0.01 M PBS and incubated with the secondary antibody Goat Anti-Rabbit IgG H&L (Alexa Fluor 488) (ab150077, Abcam, UK) in the dark for 2 h at RT. For nuclear staining, the cells were incubated with Hoechst 33342 (1:2000) for 10 min at RT and washed thrice with 0.01 M PBS. Subsequently, the coverslips were mounted on glass slides with 10 μl antifade mounting medium (Beyotime, China). Finally, the slides were visualized using a compound microscope.

### Cell viability assay

Cells were seeded in a 96-well plate at a density of 5×10^4^ cells/well in 100 μl of growth medium. After culturing in a CO_2_ incubator at 37°C for 24 hours, they were treated with or without ES, Rapa or 3-MA for 3 h and 10 μl of CCK-8 (MedChemExpress, China) solution was added to each well of the plate. After incubating the plate for 1 hour, the absorbance at 450 nm was measured using a Sunrise microplate reader (Tecan, Austria).

### Statistical analysis

Data were analysed by the GraphPad Prism 8 software for Windows. The differences between the treatment and control groups were analysed using a one-way analysis of variance (ANOVA). Data are expressed as the mean ± SD (standard deviation) of at least three repeated experiments. Values with p<0.05 were considered statistically significant. In all cases, *p*-values were expressed as *p<0.05, **p< 0.01 and ***p<0.001.

## Results

### Histopathological variations of murine diaphragms at different infection times

**Fig 1:**
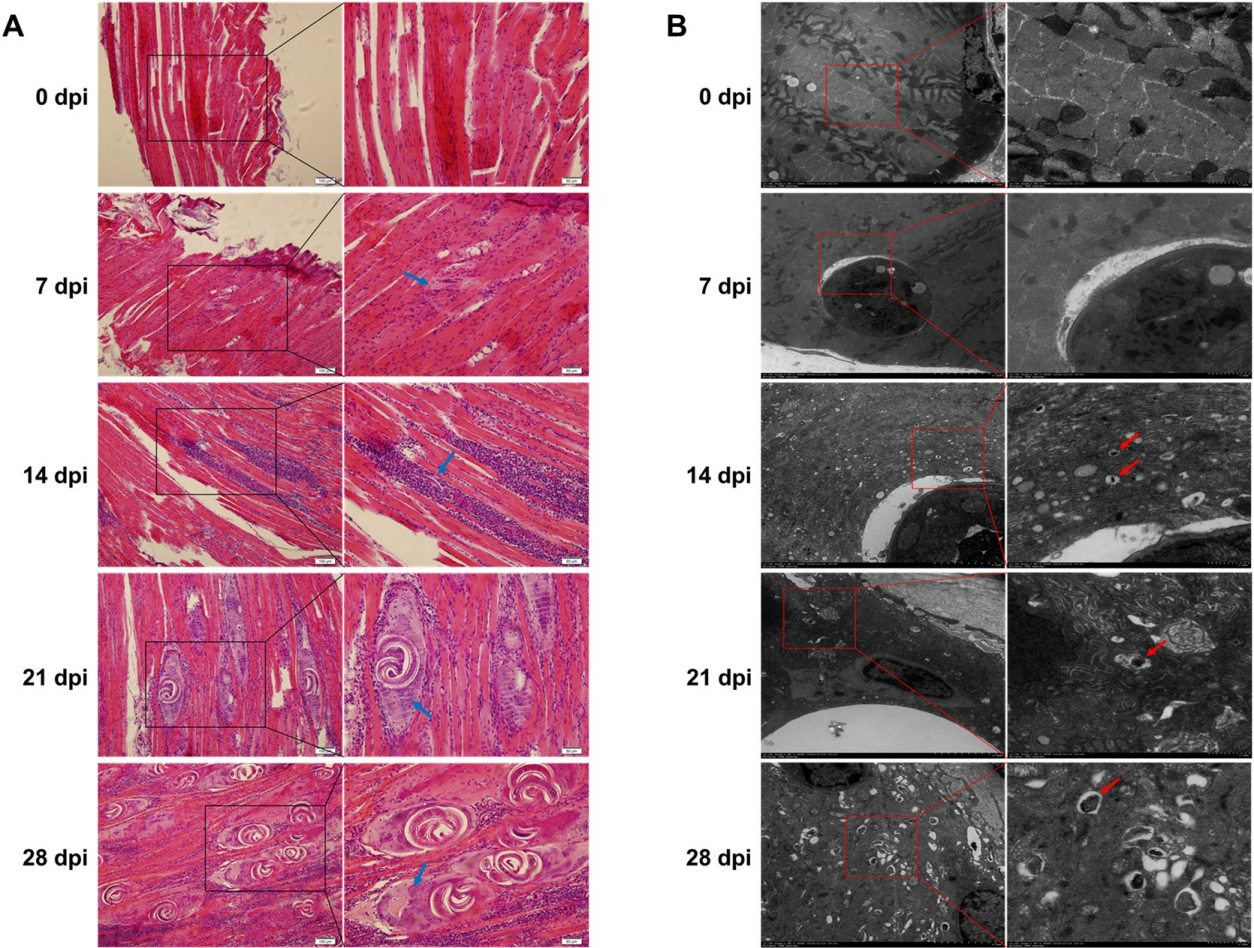
Histopathological changes and formation of autophagic-like vacuoles in *T*. *spiralis*-infected mice. A. Sections of murine diaphragms after HE staining on days 0, 7, 14, 21, and 28 post infection observed by fluorescence microscope (magnification, 200× and 400×), The arrows on 7 and 14 dpi: weeny muscle larvae, 21 and 28 dpi: enlarged muscle nuclei. B. TEM images of autophagic-like structures in diaphragms of *T*. *spiralis*-infected mice on 0, 7, 14, 21 and 28 dpi (magnification, 1.0k×, and 3.0k×). Arrows indicate autolysosome-like structures. The images are representative of 3 independent experiments.

To investigate the effect of *T*. *spiralis* infection in muscle tissues, each BALB/c mouse was inoculated with 500 infective *T*. *spiralis* larvae. Subsequently, the mice were sacrificed at 0, 7, 14, 21, 28 dpi to collect their diaphragms. After staining with hematoxylin and eosin, the muscle larvae were observed in diaphragm sections (Fig 1A). The muscle tissues maintained a normal physiological state before *T*. *spiralis* infection. However, at 7 dpi (almost the time when new-born larvae enter muscle cells), the larvae in the muscle were too tiny and indistinguishable from the muscle tissues under a high power microscope. As such, we relied on the aggregation of inflammatory cells in the muscle tissues to predict the possible trajectory of larva during its penetration into muscle cells. As the infection progressed, a few larvae surrounded by many inflammatory cells were observed in the muscle tissues at 14 dpi. On the 21 dpi, the general morphology of the nurse cells was apparent, but they still contained incompletely transformed flake-like muscle cells. Later, the nurse cell fully took shape, and many enlarged muscle nuclei were observed in the nurse cell at 21 and 28 dpi. Also, the number of inflammatory cells surrounding these larvae was significantly increased as the infection progressed.

### *Trichinella* infection can induce autophagy in mice

Transmission electron microscopy, western blot, and quantitative real-time PCR assays were performed to determine whether *T*. *spiralis* infection can trigger autophagy of the host muscle cells and the extent of autophagy. Specifically, TEM was performed to detect autophagic vacuoles in the cells, an indicator of the induction extent of autophagy. Diaphragm samples in the uninfected groups exhibited normal morphology without ultrastructural changes. In contrast, samples in the *T*. *spiralis* infected groups showed oncotic changes, as revealed by myofibers disorganization, mitochondrial swelling, and cellular lysis (Fig 1B). As the infection progressed, the number of autophagosome-like structures was significantly increased in the cytoplasm of the infected cells.

**Fig 2:**
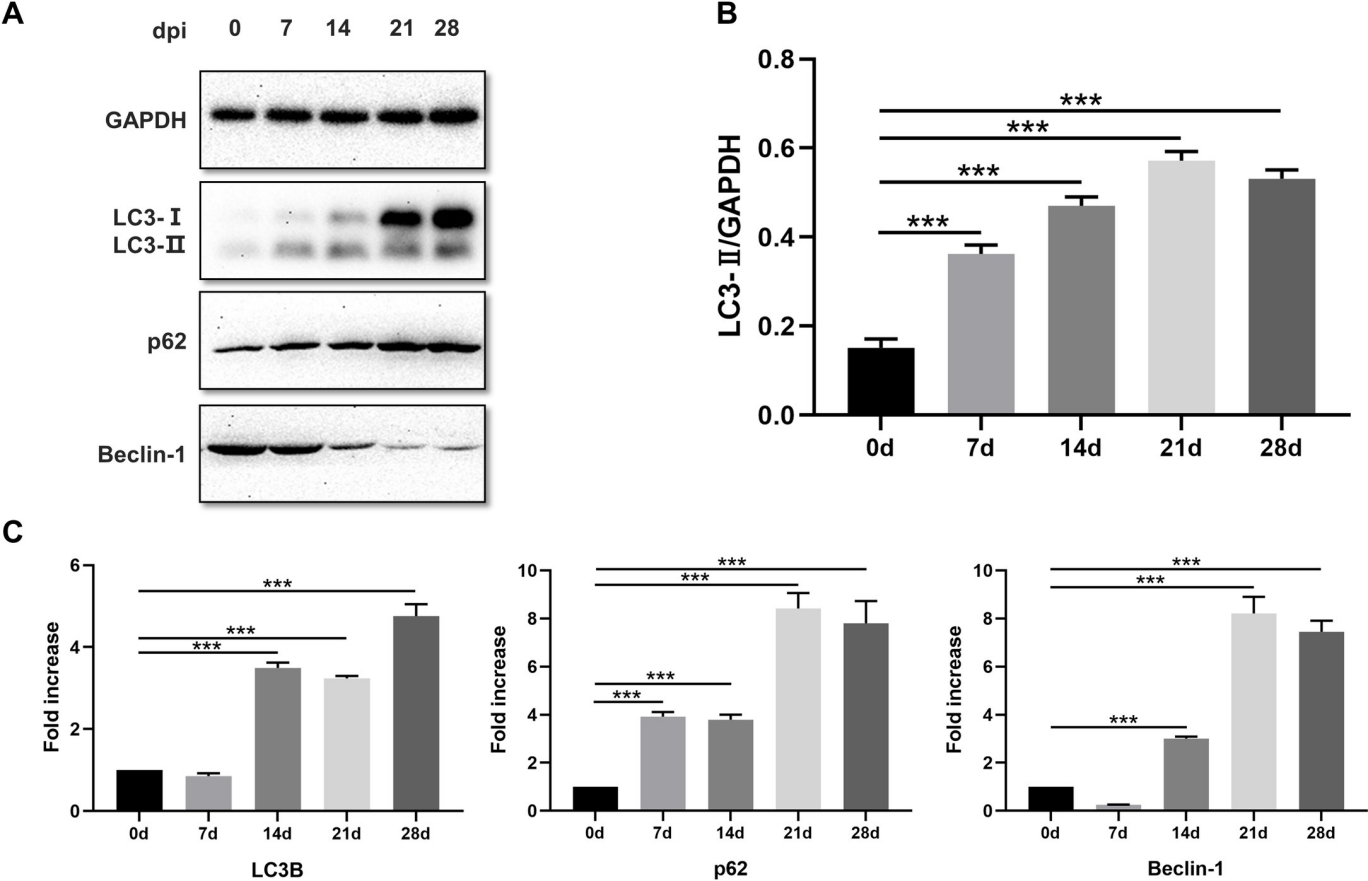
Protein level and transcriptional level of LC3B, p62, and Beclin-1 in *T*. *spiralis*-infected mice. A. The protein expression level of LC3B, p62, and Beclin-1 in diaphragms of *T*. *spiralis*-infected mice on 0, 7, 14, 21, and 28 dpi. B. Density ratios of the protein expression level of LC3-II and GAPDH on 0, 7, 14, 21, and 28 dpi. C. Expression of LC3B, p62, and Beclin-1 mRNA in diaphragms of *T*. *spiralis*-infected mice on 0, 7, 14, 21, and 28 dpi. The data represent at least 3 independent experiments and are expressed as mean ± SD. *p*<0.05(*), *p*<0.01(**), *p*<0.001(***).

To verify the results obtained via TEM, we examined the expression of autophagy specific markers LC3B, p62, and Beclin-1 at protein and mRNA levels using western blot and qRT-PCR assays, respectively. Protein expression of LC3-II in the diaphragms at 7, 14, 21, and 28 dpi increased significantly (*p*<0.001) compared with the control group and peaked at 21 days post-inoculation (Fig 2A and 2B). Contrary to the expectations, the protein expression of p62 increased while that of Beclin-1 decreased relative to the control (Fig 2A). Compared to the control group, the mRNA levels of p62 were significantly higher at all the four timepoints following infection. However, the transcriptional levels of LC3 and Beclin-1 did not differ at 7 days dpi, with both of them increasing significantly during the last three timepoints (Fig 2C). Respectively, mRNA level of these three genes were highest at 21, 28, 21 days. Although the expression levels of autophagy markers LC3-II was increased following *T*. *spiralis* infection, the expression levels of p62 and Beclin-1 were not consistent with those observed in typical autophagy.

### Changes in the transcriptional level of additional autophagy-related markers *in vivo*

**Fig 3:**
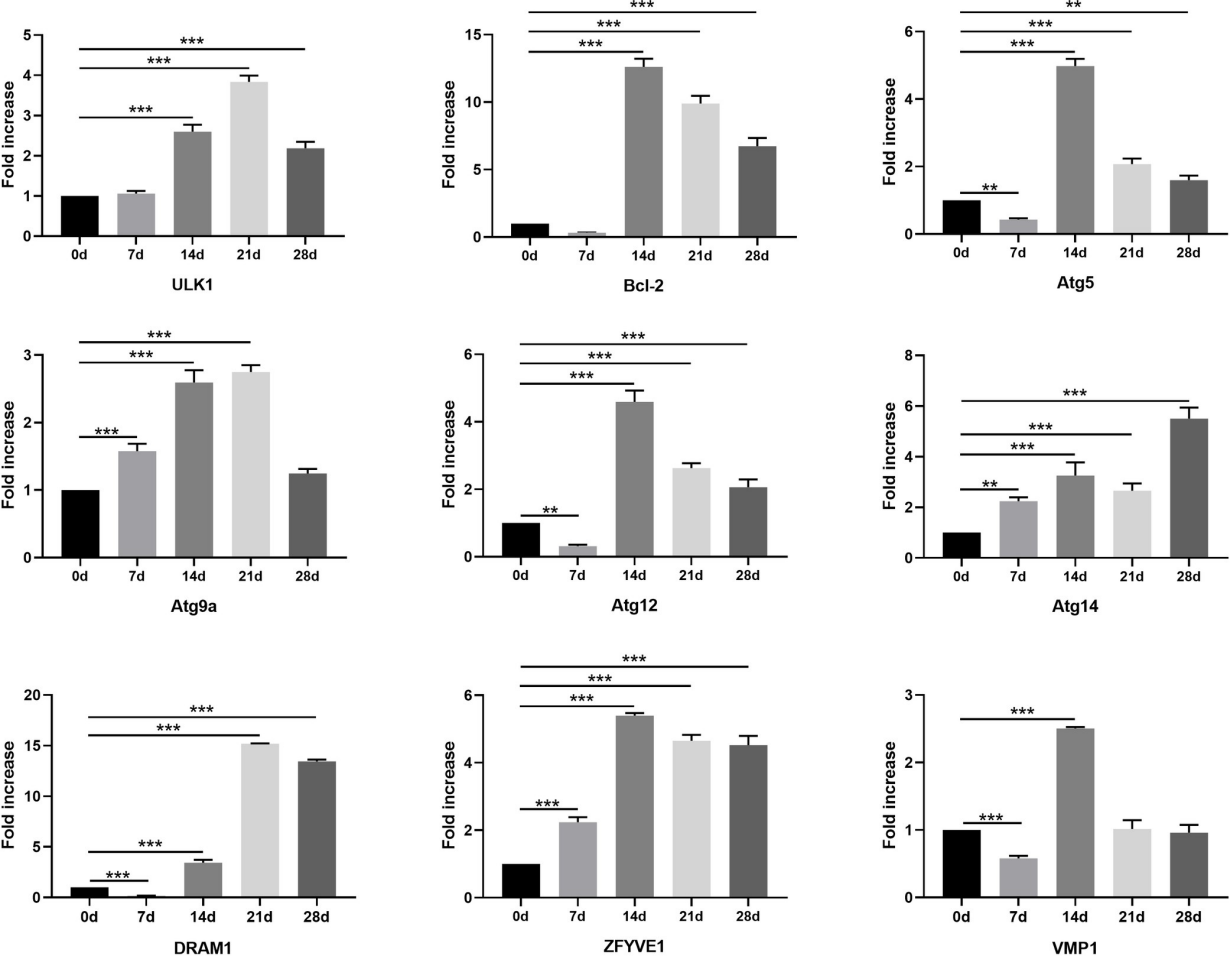
Changes in the transcriptional level of additional autophagy-related markers *in vivo*. Expression of ULK1, Bcl-2, Atg5, Atg9a, Atg12, Atg14, DRAM1, ZFYVE1, and VMP1 mRNA in the diaphragms of *T*. *spiralis*-infected mice on 0, 7, 14, 21, and 28 dpi. The data represent at least 3 independent experiments and are expressed as mean ± SD. *p*<0.05(*), *p*<0.01(**), *p*<0.001(***).

The induction of autophagy sometimes results in an increase in the mRNA levels of specific autophagy genes, including Atg7, Atg8/LC3, Atg9, Atg12, and Atg14. We extracted RNA from the diaphragms of *T*. *spiralis* infected mice at different timepoints after infection and evaluated the transcriptional levels of autophagy-related genes (Fig 3). Messenger RNA levels of the autophagy-related genes were significantly elevated at most of the timepoints after infection, except for some genes (Bcl-2, Atg5, Atg12, DRAM1, and VMP1) whose transcription levels declined at 7 dpi. Also, we found that transcriptional levels of most of the genes peaked at 14 or 21 dpi, which is the most active stage of nurse cell formation. After reaching the peak, the transcription level dropped for all the genes except Atg14, which is not localized exclusively to phagophores. Thus, monitoring changes in mRNA levels for either Atg genes or autophagy regulators may provide some evidence supporting upregulation of the potential to undergo autophagy.

### Pathways involved in the regulation of *T*. *spiralis*-induced autophagy *in vivo*

**Fig 4:**
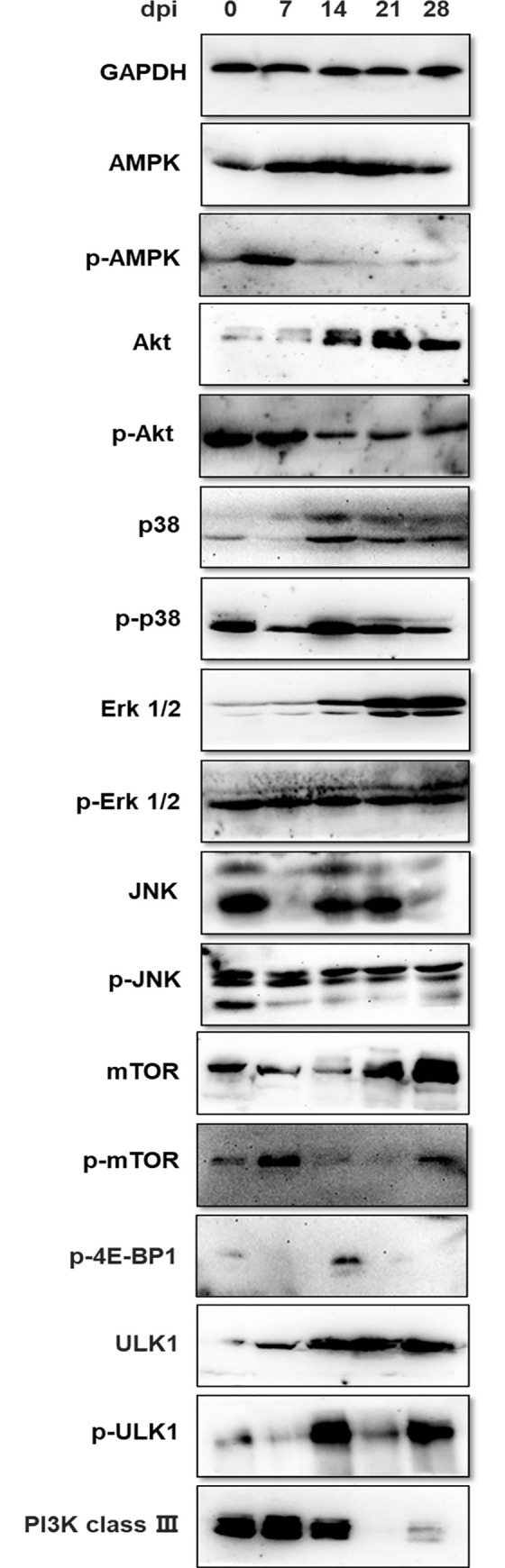
Pathways involved in the regulation of *T*. *spiralis*-induced autophagy *in vivo*. Total protein expression and phosphorylated level of AMPK, Akt, p38, Erk1/2, JNK, mTOR, 4E-BP1, ULK1, and PI3K class III in the diaphragms of *T*. *spiralis*-infected mice on 0, 7, 14, 21 and 28 dpi. The data represent at least 3 independent experiments.

To ascertain the possible role of AMPK, Akt, MAPK/JNK/Erk1/2, and mTOR pathways in *T*. *spiralis*-induced cell autophagy, we examined the expression levels of the phosphorylated forms of AMPK, Akt, JNK, p38 MAPK, Erk1/2, and mTOR (Fig 4). The expression of phosphorylated AMPK was elevated on the 7th day after the infection; however, the expression level significantly reduced at later timepoints compared to the control. Meanwhile, reduced phosphorylated levels of Akt and JNK were observed after *T*. *spiralis* infection. Besides, murine diaphragms infected with *T*. *spiralis* showed similar trends with regards to total p38 and p-p38, and exhibited lower levels at most timepoints after infection compared with the control, except at 14 dpi when their levels were elevated. Moreover, we found that *T*. *spiralis* activated total extracellular signal-regulated kinases (Erk) in a time-dependent manner *in vivo*. However, the expression of phosphorylated Erk1/2 was not changed at each timepoint. Furthermore, mTOR, an essential downstream modulator of AMPK, Akt, and MAPK/Erk1/2, showed decreased phosphorylation. Its activity can also be monitored by following the phosphorylation of 4E-BP1, a substrate of mTOR. The levels of phosphorylated 4E-BP1 were almost undetectable at all the timepoints, except at 14dpi when its levels spiked relative to the control. Meanwhile, as the core protein of the autophagy signaling pathway, total and phosphorylated levels of ULK1 were increased after infection. In contrast, the expression of PI3K class III was negligible at the late stages of the infection. These results showed that the Akt/p38 MAPK/JNK/mTOR signaling pathways could be closely linked to *T*. *spiralis*-mediated autophagy in skeletal muscle cells.

### Slight inhibition of autophagy conduced the growth of *T*. *spiralis*, whereas severe inhibition of autophagy suppressed its survival *in vivo*

**Fig 5:**
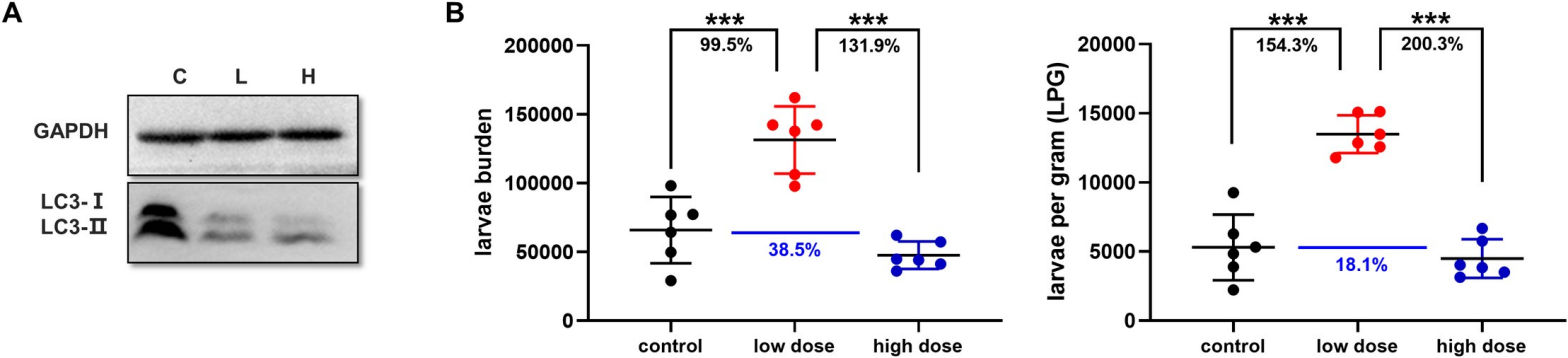
Slight inhibition of autophagy conduced the growth of *T*. *spiralis*, while severe inhibition of autophagy suppressed its survival. A. The diaphragmatic muscle LC3-II expression level after the use of autophagy inhibitor 3-MA on 28 dpi. C: control; L: low dose (5 μg/g); H: high dose (10 μg/g). B: The larvae burden and LPG of mice in the control, low-dose, and high-dose groups. Each group contained at least six mice and the data are expressed as mean ± SD. *p*<0.05(*), *p*<0.01(**), *p*<0.001(***).

Although *Trichinella* infection can lead to autophagy in murine muscle tissues, its effects on parasite survival remain unclear. To examine this, we administered the mice with different doses of autophagy inhibitor 3-MA. The expression levels of LC3-II in the diaphragm were significantly reduced following the administration of 3-MA (Fig 5A), which was more significant in the high-dose group, indicating that autophagy was successfully inhibited *in vivo*. After autophagy was inhibited, the larvae burden and larvae per gram (LPG) of the low dose group were higher than those of the normal infection group by 99.5% and 154.3%, respectively, which implied that autophagy inhibition could significantly improve the growth of parasites *in vivo* (Fig 5B). However, it should be noted that the number of worms and LPG in the high dose group was lower than that in the low dose group by 131.9% and 200.3%, control group by 38.5% and 18.1% respectively (despite not statistically significant). This result suggested that excessive inhibition of autophagy appeared to restrain parasite growth, relative to the natural infection.

### ES inhibited the autophagy of C2C12 myoblasts *in vitro*

**Fig 6:**
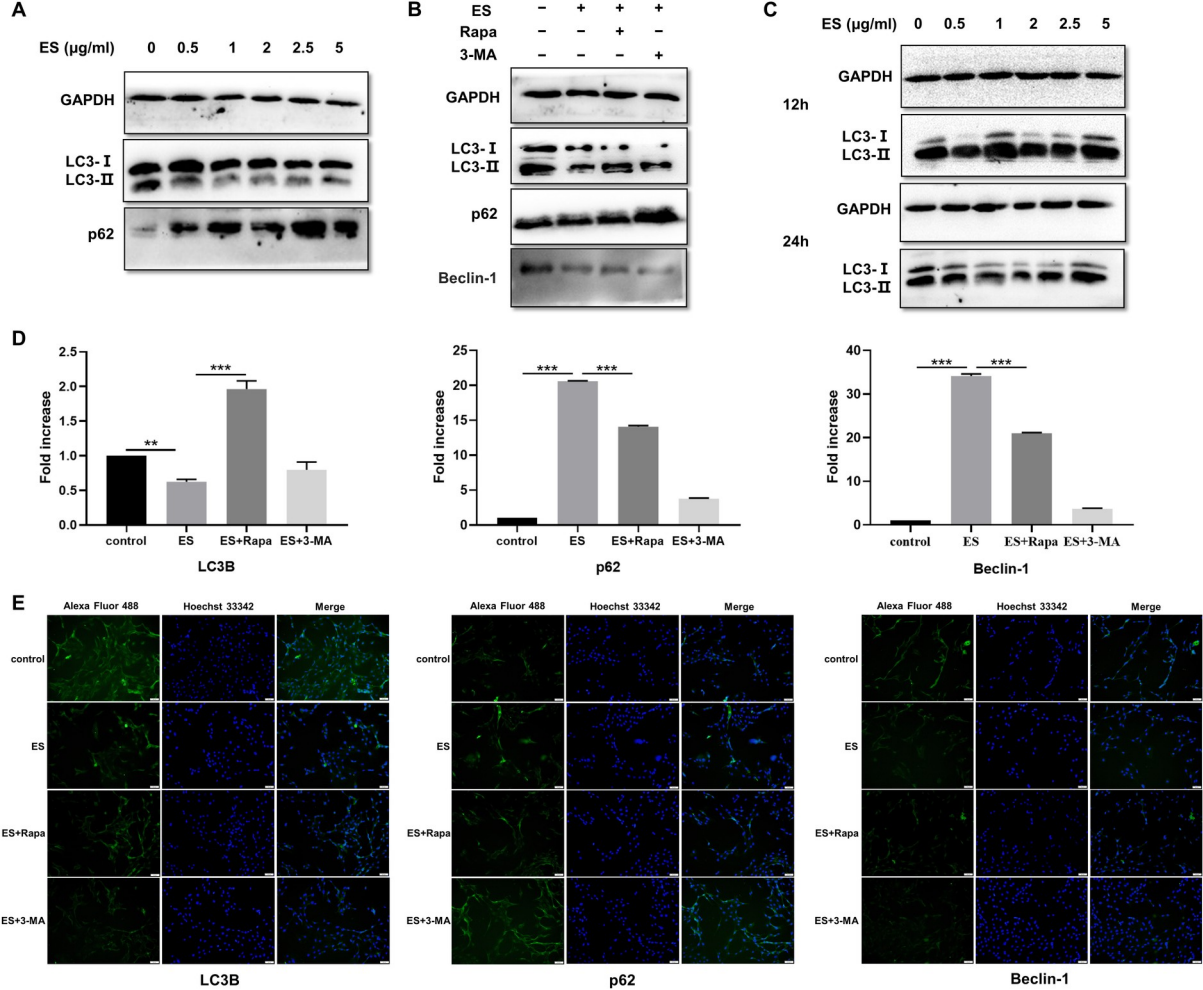
Protein level and transcriptional level of LC3B, p62, and Beclin-1 in ES-treated myoblasts. A. The protein expression level of LC3B and p62 in myoblasts treated with 0, 0.5, 1, 2, 2.5, and 5 μg/ml ES for 3 h. B. The protein expression level of LC3B and p62 in myoblasts treated with or without 1 μg/ml ES, rapamycin (100 nM), and 3-MA (5 mM) for 3 h. C. The protein expression level of LC3B and p62 in differentiated myoblasts treated with 0, 0.5, 1, 2, 2.5, and 5 μg/ml ES for 12 h or 24 h. D. Expression of LC3B, p62, and Beclin-1 mRNA in myoblasts treated with or without 1 μg/ml ES, rapamycin (100 nM), and 3-MA (5 mM) for 3 h. E. Immunofluorescence stained with LC3B, p62 and Beclin-1 antibodies in myoblasts treated with or without 1 μg/ml ES, rapamycin (100 nM), and 3-MA (5 mM) for 3 h (magnification, 400×). The data represent at least 3 independent experiments and are expressed as mean ± SD. *p*<0.05(*), *p*<0.01(**), *p*<0.001(***).

We have shown that infection of *T*. *spiralis in vivo* causes autophagy of host myocytes, but the effect of *T*. *spiralis* on autophagy of myoblasts *in vitro* deserves further verification. Myoblasts were treated with different concentrations of ES for 3 h to examine the impact of *T*. *spiralis* infection on their autophagy. The LC3-II protein expression in the treated cells was decreased in a dose-dependent manner (Fig 6A). However, the expression of p62 protein was enhanced after ES treatment and increased with the increase in ES concentration. Collectively, these results indicated that the effect of ES on the autophagy of myoblasts *in vitro* was contrary to the results of experiments conducted *in vivo*, which suggested that ES could inhibit the autophagy of myoblasts.

To determine the effects of autophagy suppression, we used a specific autophagy activator rapamycin (100 nM) and autophagy inhibitor 3-MA (5 mM) to verify the inhibition effect of ES on autophagy. Myoblasts were incubated with or without ES (1 μg/ml) for 3 h, and the protein expression levels of the autophagy-related proteins LC3-I, LC3-II, and p62 were detected using Western blot assay (Fig 6B). The expression levels of LC3-I and LC3-II were significantly decreased in the ES alone-treated group compared to the control group. Rapamycin or 3-MA influenced the expression of LC3-I, LC3-II, and p62 in ES-treated groups compared to the ES-treated alone group. Also, rapamycin increased the protein expression levels of autophagy-related proteins LC3-II and Beclin-1, whereas 3-MA reduced their protein expression levels upon ES treatment, except for p62 whose expression was significantly higher than the control.

To further verify the western blot results, we employed q-PCR and cellular immunofluorescence staining assays to examine the expression of autophagy-related proteins in the four groups. The mRNA levels of p62 and Beclin-1 in the ES-treated groups were significantly upregulated compared to the untreated groups. Notably, the transcriptional levels of LC3B were consistent with the reduced protein expression levels (Fig 6D). Immunofluorescence results (Fig 6E) showed that the fluorescence intensity of LC3B and Beclin-1 significantly decreased after ES treatment compared with the control group, rapamycin and 3-MA also showed the same promoting or antagonistic effects on autophagy upon ES treatment as western blot results. Moreover, the strongest fluorescence intensity of p62 was observed in the ES+3-MA-treated group. Taken together, these results suggested that ES might inactivate the mechanism of autophagy induction. The degree of inhibition could also be affected by autophagy agonists or inhibitors.

Besides, differentiated myoblasts were used to determine whether ES influenced its autophagy. C2C12 myoblasts were induced to differentiate in the differentiation medium consisting of DMEM supplemented with 2% (v/v) horse serum for five days. The differentiated myoblasts were treated with different concentrations of ES for 12 or 24 h after the formation of the myotube (Fig 6C). Western blot results showed that although the expression level of LC3-II protein was discrepant after treatment with different concentrations, there was no apparent variation tendency, and the decreased protein expression was only noticed at 24 h after treatment with low concentrations of ES. These results implied that ES did not exert uniform effects on the autophagy of differentiated myoblasts.

### Variations in the transcriptional level of additional autophagy-related markers *in vitro*

**Fig 7:**
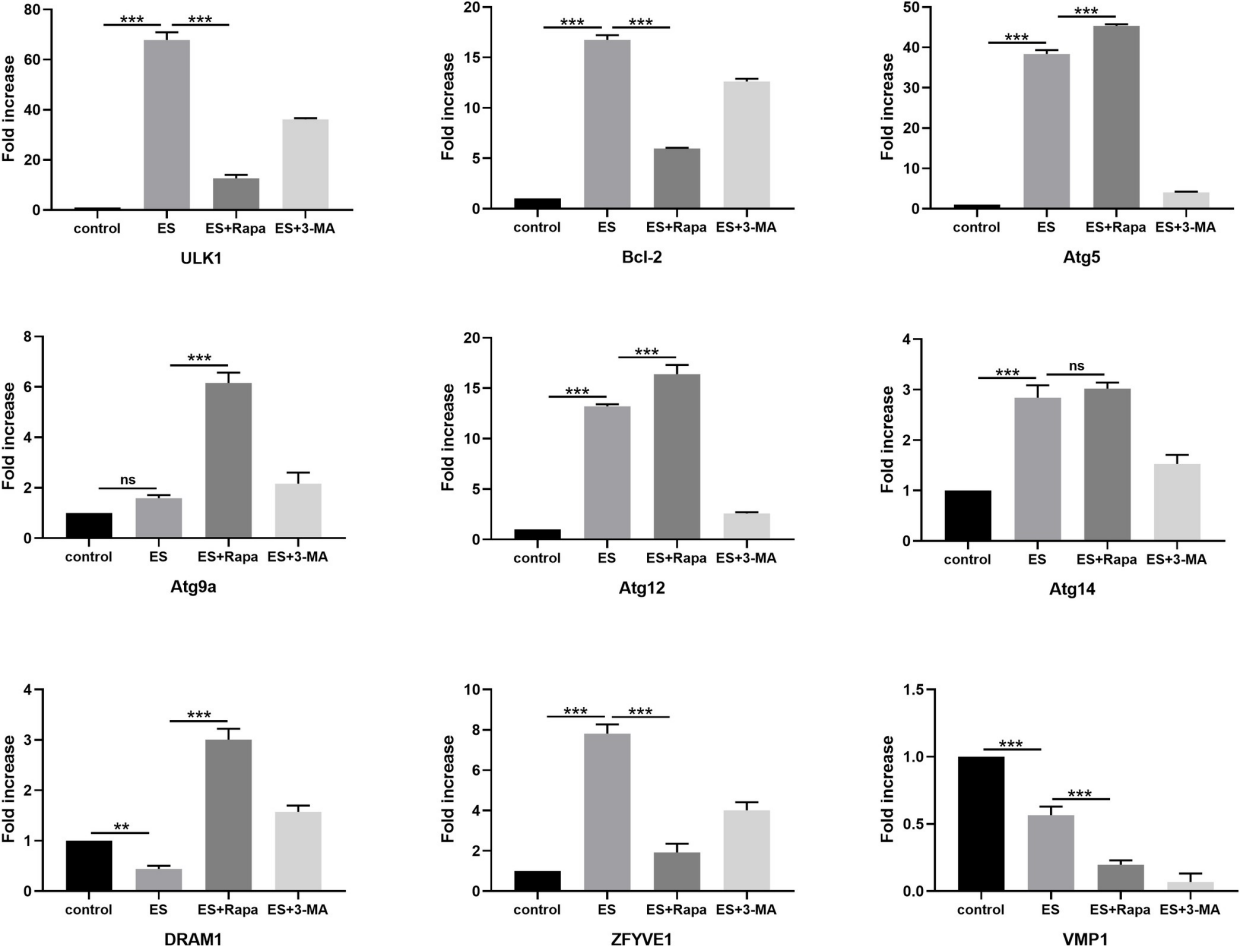
Changes in transcriptional level of additional autophagy-related markers in ES-treated myoblasts. Expression of ULK1, Bcl-2, Atg5, Atg9a, Atg12, Atg14, DRAM1, ZFYVE1, and VMP1 mRNA in myoblasts treated with or without 1 μg/ml ES, rapamycin (100 nM), and 3-MA (5 mM) for 3 h. The data represent at least 3 independent experiments and are expressed as mean ± SD. *p*<0.05(*), *p*<0.01(**), *p*<0.001(***), ns = no significance.

Cellular RNA of different groups was also extracted for transcriptional level analysis (Fig 7). The mRNA levels of most autophagy-related genes were elevated upon ES treatment, and only two genes (DRAM1 and VMP1) had lower transcription levels than myoblasts in the control groups. After the addition of autophagy inducer Rapa, gene expression levels of Atg family members increased compared with the ES-treated alone group. Regarding the co-treatment with 3-MA, the expression levels of most autophagy-related genes were significantly decreased compared with the ES-treated alone group. Unlike the results of *in vivo* experiments, changes in the mRNA expression levels of autophagy-related genes were reversed with the inhibiting autophagy following ES treatment.

### Pathways involved in the regulation of ES-inhibited autophagy *in vitro*

**Fig 8:**
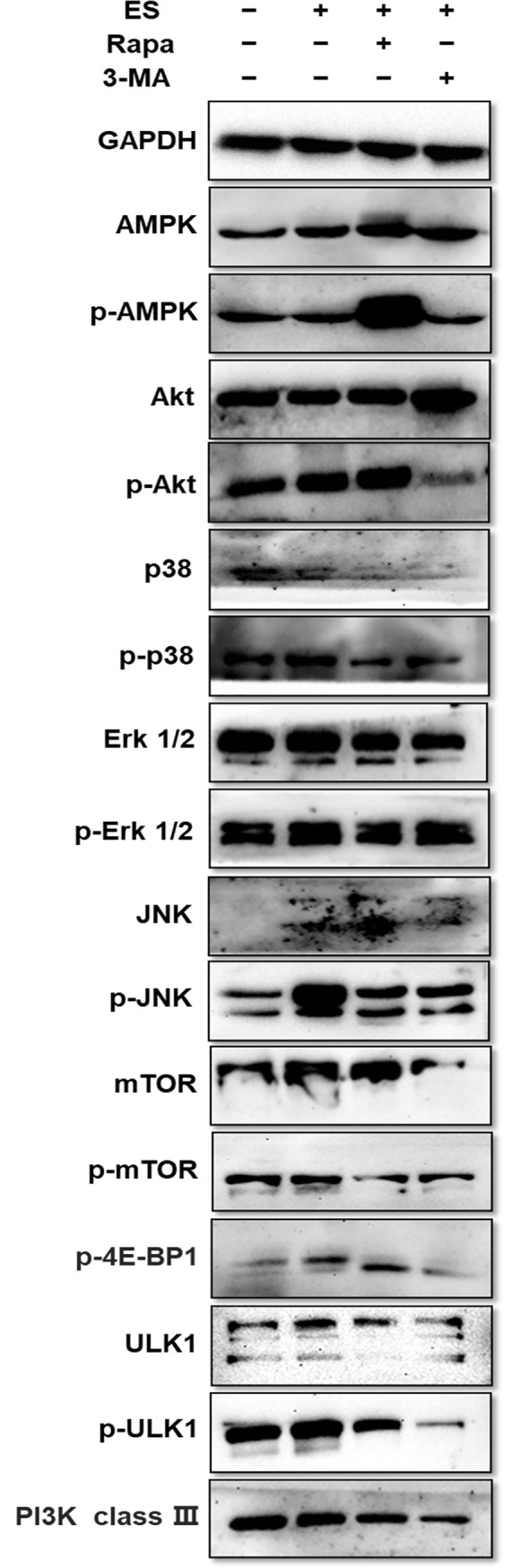
Pathways involved in the regulation of *T*. *spiralis*-induced autophagy in ES-treated myoblasts. Total protein expression and phosphorylated level of AMPK, Akt, p38, Erk1/2, JNK, mTOR, 4E-BP1, ULK1, and PI3K class III in myoblasts treated with or without 1 μg/ml ES, rapamycin (100 nM), and 3-MA (5 mM) for 3 h. The data represent at least 3 independent experiments.

Since we have detected pathways involved in the regulation of *T*. *spiralis*-induced autophagy *in vivo* above, pathways involved in the regulation of ES-inhibited myoblasts autophagy also deserves further investigation. To further explore the mechanism by which ES inhibited autophagy of myoblasts, additional experiments were conducted (Fig 8). Although the levels of phosphorylated AMPK were slightly lowered by ES treatment, the total protein expression of AMPK and mTOR increased. No significant change was observed regarding the phosphorylation of mTOR. However, an upward trend in the phosphorylation level of its substrate 4E-BP1 was observed after ES treatment. Meanwhile, the expression of phosphorylated forms of Akt, p38, JNK, and Erk1/2 increased in the ES treated group compared with the untreated group. Besides, after ES treatment, the phosphorylation level of ULK1 increased, but that of PI3K class III decreased slightly. These results showed that Akt/p38 MAPK/JNK pathway might be involved in ES-inhibited autophagy in myoblasts.

### No significant effect of ES-suppressed autophagy on myoblasts viability

**Fig 9:**
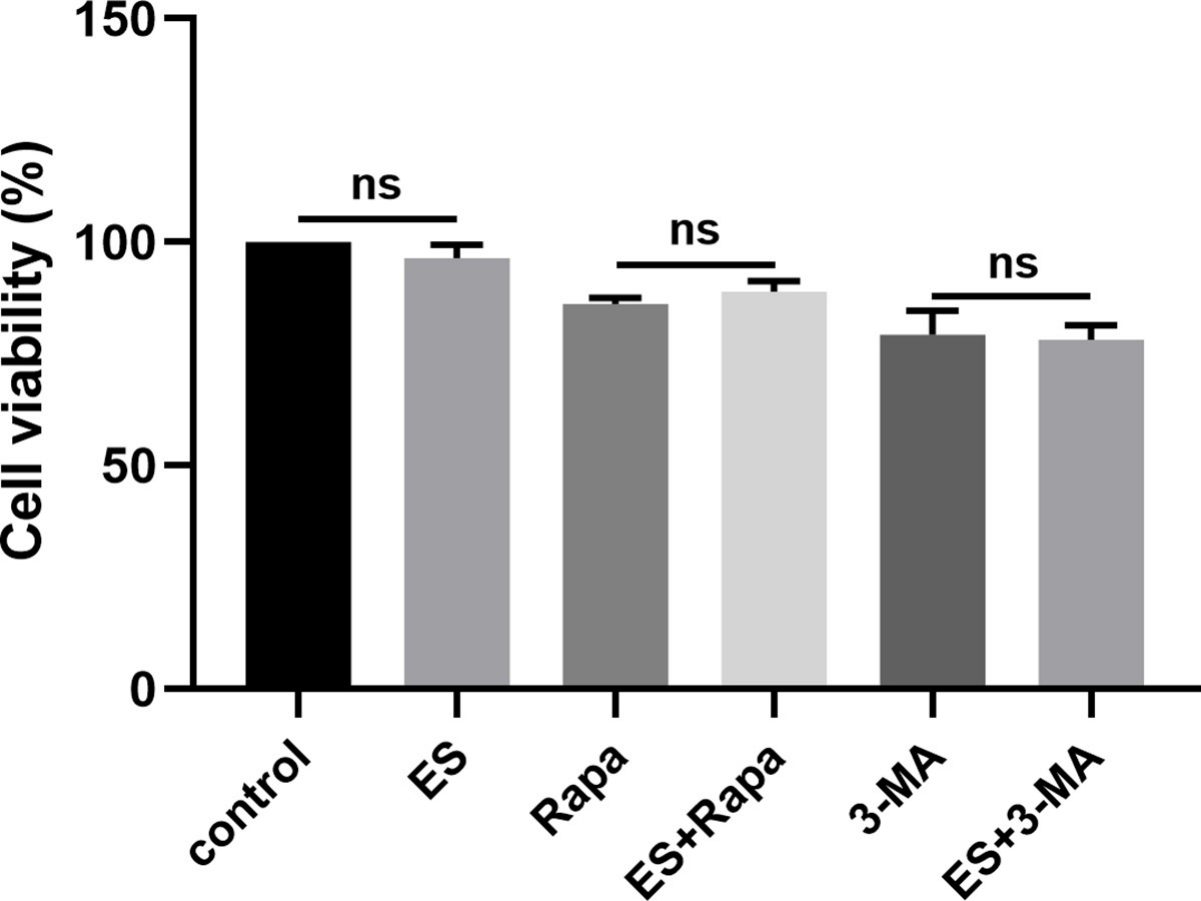
No significant effect of ES-suppressed autophagy on myoblasts viability. Cell viability was detected using the CCK8 assay following that C2C12 myoblasts were treated with or without 1 μg/ml ES, rapamycin (100 nM), and 3-MA (5 mM) for 3 h. The data represent at least 3 independent experiments and are expressed as mean ± SD. ns = no significance.

Previous studies have shown that *Angiostrongylus cantonensis* ES could reduce cell survival. Also, the use of autophagy activator has been shown to enhance cell viability through the activation of the autophagy mechanism in astrocytes [15]. Given that ES of *T*. *spiralis* restrained intracellular autophagy in myoblasts, we speculated that it could affect the survival rate of myoblasts. To investigate the effects of autophagy in ES-treated myoblasts, cells were pretreated with an autophagy activator or inhibitor and incubated with ES for 3 h. Our first objective was to determine the effect of ES on myoblasts. Cells were pretreated with or without 1 μg/ml ML-ES, and cell viability was evaluated using the CCK8 assay. Based on the assay, ES treatment slightly lowered the viability of myoblasts, but the effect was not statistically significant (Fig 9), indicating that ES inhibition could not reduce cell activity. Also, we further examined whether autophagy inactivation can protect myoblasts upon drugs treatment. Cells were pretreated with autophagy activator or inhibitor. According to the results, rapamycin or 3-MA reduced cell viability of myoblasts. However, treatment with ES did not alter the decreased cell activity caused by drugs. And activation or inhibition of autophagy could all result in reduced cell activity, compared with cells treated with ES alone. These results demonstrated that ES-induced autophagy inhibition might not affect the viability of myoblasts.

## Discussion

Numerous studies demonstrated that autophagy could be triggered during microbial infection, such as bacteria, viruses, protozoa, and parasitic helminths [20–23]. However, autophagy induction has not been confirmed in *Trichinella* infection. The present study examined the relationship between autophagy and *T*. *spiralis* infection or ES treatment. Our results showed that *T*. *spiralis* infection could induce the occurrence of autophagy in the diaphragms of mice. Infiltration and aggregation of inflammatory cells were observed in the process of NBL penetrating into muscle cells to develop into ML through blood or lymph fluid, scilicet the process of nurse cell formation, and TEM also revealed the formation of autophagosomes. Moreover, we conducted western blot and qPCR assays using the protein and RNA extracted from the murine diaphragms to confirm further whether the parasitic infection induced autophagy. Increased expression of autophagy marker LC3-II was detected at the protein and transcription levels.

Considering that *T*. *spiralis* could induce autophagy in the host, we hypothesized that ES might be involved in autophagy induction. Thus, we treated C2C12 cell lines with different concentrations of ML ES. Treatment with ES inhibited the autophagy of myoblasts. Concerning the levels of protein expression, LC3-II was significantly downregulated, while p62 was upregulated after ES treatment. To verify these results further, we examined the expression of autophagy markers through cellular immunofluorescence and qPCR assays, which revealed similar findings. Collectively, these findings indicated that ES could inhibit the occurrence of autophagy in myoblasts.

In addition to LC3 (the protein mostly used for monitoring autophagy), other markers can also be used to confirm further that *T*. *spiralis* infection induced autophagy. Here, we selected some of the most commonly used or better-characterized LC3 alternatives. P62 and p62-bound polyubiquitinated proteins get incorporated into the completed autophagosome and are degraded in autolysosomes. Thus, this protein can serve as an indicator of autophagic degradation. The inhibition of autophagy correlates with increased levels of p62 in mammals, suggesting that steady-state levels of this protein can reflect the autophagic status [24]. Whereas, there is not always a clear correlation between increases in LC3-II and decreases in p62. For example, p62 upregulation and at least transient increases in the amount of p62 is seen in some situations, in which there is an increase in autophagic flux [25]. The same phenomenon was observed in *T*. *spiralis* infection *in vivo*, despite we monitored the p62 mRNA level else as part of a complete analysis. Beclin-1 is essential in the autophagy interactome that signals the onset of autophagy and has been extensively used to monitor autophagy [26]. Binding of the protein to the anti-apoptotic protein Bcl-2 inhibits its activity, and autophagy is induced by the release of Beclin-1 from Bcl-2 [27]. However, it is essential to note that certain forms of macroautophagy are induced in a Beclin-1-independent manner [28]. Moreover, it is noteworthy that caspase cleavage of Beclin-1 can be detected under standard culture conditions [29], which could explain why the autophagy caused by *T*. *spiralis* infection was accompanied by a decrease in Beclin-1 protein expression in this study. Atg5–Atg12 conjugation has been used in some studies to measure autophagy. It appears essentially that all the Atg5 and Atg12 proteins exist in the conjugated form and their expression levels do not change in mammalian cells [30], and this was also consistent with our qPCR results. Atg9 is the only integral membrane Atg protein that is essential for autophagosome formation in all eukaryotes. Atg14 and ZFYVE1 localize primarily in phagophores. In addition to the Atg genes, transcriptional upregulation of VMP1 can be detected in cells undergoing autophagy [31]. The elimination of DRAM1 by siRNA blocks autophagy, indicating that it is an essential component of the process [32]. All the genes mentioned above can be modulated to increase in the transcriptional level at a specific or several timepoints during nurse cell formation. It is further believed that *T*. *spiralis* infection induced autophagy.

Given that the results of *in vivo* infection versus ES treatment *in vitro* were contradictory, we further explored the pathways involved in the regulation of these events. mTOR is a central autophagy-suppressive regulator that integrates growth factor, nutrient, and energy signals. In most systems, inhibition of mTOR leads to the induction of autophagy. Meanwhile, AMPK activity is generally antagonistic toward mTOR function. AMPK has been shown to stimulate autophagy in many cell types, including fibroblasts, colon carcinoma cells, and skeletal muscles [33]. The mTOR pathway is activated by upstream kinases, such as AKT and MAPK, under sufficient energy supply. The classical MAPK pathway consists of the Erk and the c-jun N-terminal kinase (JNK) [34]. AMPK and mTOR regulate autophagy through coordinated phosphorylation of ULK1, whose kinase activity increases when autophagy is induced, irrespective of the pathway regulating the induction. The identification of ULK1 as a direct target of mTOR and AMPK was a significant step towards the development of new tools for monitoring autophagy induction. Furthermore, ULK1 can mediate the inhibitory phosphorylation of AMPK to generate a negative feedback loop [35]. This may be the reason why there was no significant change in the AMPK pathway after ES treatment in this study. Likewise, mTOR activity can be monitored by following the phosphorylation of its substrates 4E-BP1. Herein, we analyzed the phosphorylation of 4E-BP1 at Thr37 and Thr46, which are directly phosphorylated by mTOR [36]. Within the autophagy network, multiple class III PI3-kinase complexes, such as Beclin-1, are required for the induction of autologous phagocytosis in eukaryotes [37]. According to the results of our *in vitro* and *in vivo* experiments, the pathways with the most significant change were Akt/p38 MAPK/JNK pathways, which were speculated to be involved in the regulation of *T*. *spiralis*-induced and ES-suppressed autophagy. In this study, the expression of phosphorylated AMPK did not change significantly *in vitro* and *in vivo*, except for one time point *in vivo*. Although the phosphorylation of mTOR decreased *in vivo*, there was no significant change *in vitro*; however, the phosphorylation level of its substrate 4E-BP1 was increased. In addition, the results obtained from the detection of these pathways can also be instructive for the *T*. *spiralis* research in other fields related to these pathways, for instance, proliferation, differentiation and apoptosis.

Autophagy has opposing roles in cancer and interventions to both stimulate and inhibit autophagy have been proposed as cancer therapies. The pro-tumorigenic effects are generally believed to result from the ability of autophagy to sustain the survival of tumor cells during metabolic stress by providing nutrients to the malignant cells via the stromal cells in the tumor microenvironment [38]. Once cancer is established, increased autophagic flux often enables tumor cell survival and growth [39]. Conversely, recent studies have demonstrated that autophagy could play a role in both cancer and bone marrow cells to suppress tumorigenic inflammation and enhance adaptive immunity that inhibits cancer growth and progression [40,41]. Interestingly, *T*. *spiralis* and its ES have shown dual effects on tumor development. Infection by *T*. *spiralis* can inhibit tumor growth and prolong host survival time. In 1970, Weatherly was the first to report that the administration of sublethal infections of *T*. *spiralis* muscle larvae significantly increased the survival rates of Swiss mice with breast tumors. Also, the antitumor activity of the cytotoxic factor was induced through apoptosis in mice thymocytes by *T*. *spiralis* ES products [42]. While coinfection of *T*. *spiralis* with cancer and Th2-polarized responses induced by *Trichinella* infection may not have an antitumor effect but promote tumor occurrence or recurrence [43]. However, further studies should be conducted to examine whether the dual effect exerted by *T*. *spiralis* and its ES products on autophagy can be utilized in the prevention or inhibition of tumors.

The results of the present study provided a compelling explanation of the dual-action. In this study, two contrary effects were observed in *T*. *spiralis* infection and ES stimulation on myoblasts *in vitro*; that is, *in vivo* promotion of autophagy and *in vitro* inhibition of autophagy. Although autophagy can degrade intracellular pathogens, *T*. *spiralis* could be too large to wrap. Besides, our microscopy results did not show structures resembling autophagic vacuoles engulfing whole parasites. Meanwhile, we discovered that slight inhibition of autophagy conduced the growth of *T*. *spiralis*. In contrast, severe inhibition suppressed its survival *in vivo*, and this was consistent with the results observed in *Toxoplasma gondii* and *Plasmodium* studies [13,14]. Contrary to the implication of host autophagy in the control of intracellular parasites, parasites can also antagonize the host autophagy machinery, or even exploit it to enhance their development.

Not only that, our research results are more in-depth on the basis of previous achievements. We used ES to treat muscle cells and obtained the opposite results to *in vivo* experiments. Interestingly, *in vitro* results showed that ES inhibited autophagy. Strikingly, mild inhibition of autophagy is beneficial for the survival of parasites. Based on the above results, we hypothesized that the host autophagy induced by *T*. *spiralis* infection could be a defensive reaction against parasites invasion. To better colonize the host, parasites secrete ES which inhibited the autophagy of muscle cells, and use autophagy to promote *T*. *spiralis* to develop. However, this inhibition did not affect the proliferation of myoblasts, suggesting that *T*. *spiralis* might have evolved to balance the effects of parasites-induced autophagy between parasites development and host survival. The secretion of ES by the parasite could be an adaptation to maintain host cell autophagy at a level that is conducive for parasite growth and development. In addition, the opposite results *in vitro* and *in vivo* are possibly due to not only the role of *T*. *spiralis* and its ES products, but also the involvement of immune cells *in vivo*. However, inhibition of autophagy is the result of ES acting solely on muscle cells *in vitro*, which requires further investigation.

Additionally, a study reported that suppressing autophagy with various inhibitors during differentiation interferes with myogenic differentiation, highlighting the integral role of autophagy and more specifically mitophagy in myogenic differentiation [44]. The excretory-secretory products released by the *Ts*-ML have been suggested to play a crucial role in the formation of nurse cell. Furthermore, specific critical mediators contained in ML-ESP inhibited myogenesis by facilitating the proliferation of skeletal myoblasts. Also, these mediators suppressed myoblasts differentiation by inhibiting the phosphorylation of p38 MAPK in differentiating C2C12 myoblast [45–47]. The results of ES inhibiting autophagy in myoblasts we obtained can also explain its inhibition of differentiation, providing insight into the possibility that autophagy mechanism utilised by *T*. *spiralis* to affect nurse cell formation. We are focusing on the most common autophagy in this study, and further investigation on mitochondrial autophagy should be conducted. Previous studies have suggested that the immune system was induced in response to the predominance of a Th2 response during the muscle stage of *T*. *spiralis* infection. The expression of IL-4, IL-10, TGF-β and IL-13 increased during the muscle stage [48,49]. Moreover, IL-10 and TGF-β have been demonstrated to induce autophagy [50,51]. Immunoregulatory effects between host autophagy and *Trichinella* is worth pondering and large-scale specially implemented to conduct in-depth research in future studies. Despite these promising prospects, this study still has some deficiencies. Whether the infection of *T*. *spiralis* causes autophagy in the muscle stage was only examined in the present study. While the host autophagy in the intestinal stage, and in vitro studies on the relationship between adult worms ES with autophagy in small intestinal cells deserves further study. In this way, the host autophagy status at all stages of *Trichinella* infection can be completely described. In addition, synchronous infection with NBL may make the characteristics of autophagy in muscle phase more obvious, which may also contribute to a deeper understanding of host autophagy caused by *Trichinella*.

It can be seen from this study that host autophagy induced by *Trichinella* infection can eliminate parasites, and the use of appropriate autophagy inhibitors can promote *T*. *spiralis* growth. This provides the possibility and insight for utilizing autophagy to treat trichinellosis and other parasitic diseases by inducing applicable autophagy in the host. Additionally, host autophagy has been reported to help regulate of innate immunity and enhance antigen presentation in adaptive immunity [9]. Therefore, stimulating this pathway by pharmacological drugs has potential therapeutic significance for the prevention and treatment of infectious diseases [52]. There have been extensive preclinical animal model data supporting the therapeutic effect of autophagy upregulation in several diseases [53]. In conclusion, the present study firstly set forth effects of *T*. *spiralis* and ES products on autophagy of host muscle cells *in vivo* and *in vitro*, and put forward views on how *T*. *spiralis* might exploit host cell autophagy for its survival. Research on the action pathways on autophagy by parasites may contribute to the elucidation of complex mechanisms involved in cell-parasite interactions during *T*. *spiralis* infection. Moreover, further studies are necessary to clarify the specific mechanism of *T*. *spiralis* affecting each stage of the host cell autophagy process.
